# An emerging role for adenosine and its receptors in bone homeostasis

**DOI:** 10.3389/fendo.2012.00113

**Published:** 2012-09-18

**Authors:** Jack Ham, Bronwen A. J. Evans

**Affiliations:** ^1^ Institute of Molecular and Experimental Medicine, School of Medicine, Cardiff UniversityCardiff, Wales, UK; ^2^ Cardiff Institute of Tissue Engineering and Repair, School of Medicine, Cardiff UniversityCardiff, Wales, UK

**Keywords:** bone, adenosine, adenosine receptors, osteoclasts, osteoblasts, mesenchymal stem cells

## Abstract

Bone is continually being remodeled and defects in the processes involved lead to bone diseases. Many regulatory factors are known to influence remodeling but other mechanisms, hitherto unknown, may also be involved. Importantly, our understanding of these currently unknown mechanisms may lead to important new therapies for bone disease. It is accepted that purinergic signaling is involved in bone, and our knowledge of this area has increased significantly over the last 15 years, although most of the published work has studied the role of ATP and other signaling molecules via the P2 family of purinergic receptors. During the last few years, however, there has been increased interest within the bone field in the role of P1 receptors where adenosine is the primary signaling molecule. This review will bring together the current information available in relation to this expanding area of research.

## PURINERGIC SIGNALING AND BONE

Bone is a dynamic living tissue that undergoes continuous and life-long remodeling. This process is orchestrated by the three bone cell types (osteoclasts, osteoblasts, and osteocytes) and is modulated by many factors including mechanical forces and soluble osteogenic and osteolytic compounds. Impairments in the remodeling processes eventually lead to diseases such as osteoporosis. These bone diseases are major health problems and especially affect specific groups of people, e.g., elderly individuals, patients treated with corticosteroids. Furthermore, arthritis (e.g., rheumatoid arthritis, osteoarthritis) results in a heavy toll on bone, particularly in relation to bone mineral density. Although there is currently much insight into the systems that maintain healthy bone, there is still a need to develop new therapies.

It is well recognized that purines act as extracellular signaling molecules by binding to and activating the P2 and P1 (or A) family of purinergic receptors (Burnstock, 2011). These signaling cascades are known to play key roles in both neuronal and non-neuronal tissues, and there has, over the last 15 years, been increasing interest in the specific functions of purinergic signaling in the physiology and pathophysiology of bone. Much of this reported work has investigated the specific role of ATP and P2 receptors in bone homeostasis (Reyes et al., 2011; Gartland et al., 2012). Up until 6 years ago, however, very little was known about the role of P1 (or A) receptors in bone. This review will focus on recent developments in this research field and will include effects on mature osteoblasts and osteoclasts as well as their respective precursor cells. We are not aware of any studies on the expression and function of adenosine receptors (ARs) in osteocytes and these cells will not be included further in this review.

## ADENOSINE METABOLISM AND ADENOSINE RECEPTORS

**FIGURE 1:**
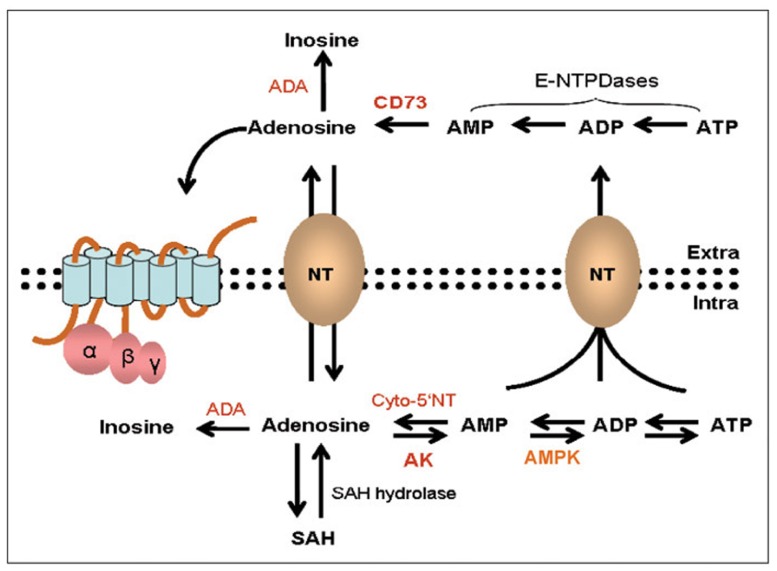
**Adenosine synthesis and metabolic pathways inside and outside of a cell.** Within the cell ATP and adenosine are continually recycled depending on energy requirements via a series of dephosphorylation and phosphorylation steps mediated by enzymes such as cytosolic 5′-nucleotidase (cyto-5′NT), adenosine kinase (AK), and AMP kinase (AMPK). Under physiological conditions the rate limiting enzyme is AK. Adenosine can also be generated from the hydrolysis of *S*-adenosyl-homocysteine (SAH). In response to high metabolic activity ATP and adenosine is extruded to the outside of the cell via facilitated diffusion and secondary active transport. Under such conditions there is further degradation of ATP to adenosine where it can activate adenosine receptors. Excess adenosine is irreversibly deamidated to inosine by the enzyme adenosine deaminase (ADA). Modified with permission from Blackburn (2003).

Nucleotides such as ATP, ADP, GTP, and GDP occur in high concentrations (2–5 mM) within cells (Hoebertz et al., 2003; Evans et al., 2006). The half-life of such molecules is, however, very short and they are sequentially degraded to the corresponding nucleoside by a family of phosphatases (e.g., ATP is sequentially dephosphorylated to ADP), AMP and adenosine are then recycled back to ATP by a series of specific kinases in a continuous cycle of ATP breakdown and formation depending on the energy requirements of the cell (Collis and Hourani, 1993; Olah and Stiles, 1995; Spychala, 2000; Fredholm et al., 2011; **Figure 1**). Under physiological conditions adenosine concentrations inside of the cell are relatively low (<1 μM) and the major regulatory enzyme is adenosine kinase (AK) which mediates the initial phosphorylation step, via AMP, back to ATP (Synder and Lukey, 1982; Pak et al., 1994; Lloyd and Fredholm, 1995; Fredholm, 2010).

The recycling of adenosine and ATP is perturbed during conditions of metabolic stress, high cellular activity or cell death, and the increased phosphatase activity leads to excessive levels of adenosine (Spychala, 2000). In addition there is increased facilitated diffusion and secondary active transport of nucleotides out of the cell where the predominant enzyme activity is ecto-5′-nucleotidase (CD73) leading to an accumulation of extracellular adenosine (**Figure 1**) where it can activate the P1 (adenosine) family of membrane bound receptors (Van Belle et al., 1987; Latini et al., 1999; Lewis et al., 2006). In contrast to intracellular adenosine which is under the regulation of AK, extracellular adenosine levels are critically regulated by adenosine deaminase (ADA) which degrades adenosine to inosine (Spychala, 2000). Another key enzyme that is important in the generation of ATP in the ATP–adenosine pathway is AMP-activated protein kinase (AMPK). AMPK is a serine–threonine kinase that regulates energy homeostasis and responds to diet and exercise (Jeyabalan et al., 2012).

The *K*_m_ (Michaelis constant denoting the substrate concentration at which the reaction rate is at half-maximum) value for ADA is much higher (70 μM) than that for AK (40 nM) indicating that AK is activated at much lower concentrations of adenosine compared to ADA (Spychala et al., 1996; Spychala, 2000). The relatively high *K*_m_ value for ADA suggests it may act as a switch for removing excessive extracellular adenosine. Thus the ATP metabolic pathways within and outside of the cell are quite different; ATP–adenosine interconversion is maintained intracellularly by cytosolic 5′-nucleotidase and AK, and extracellular ATP is converted to adenosine and inosine by CD73 and ADA (Sala-Newby et al., 1999; Bianchi and Spychala, 2003; Hunsucker et al., 2005). The relative activities of CD73 and ADA will also dictate whether a cell has a net adenosine-producing phenotype; enzyme levels however can and do fluctuate, e.g., raised adenosine levels in hypoxia appear to be due to increased CD73 expression (Spychala, 2000; Eltzschig et al., 2003). Adenosine can also be generated through the metabolism of the amino acid, L-homocysteine via the enzyme, *S*-adenosyl-homocysteine (SAH) hydrolase; this enzyme mediates the conjugation of L-homocysteine and adenosine when the amino acid levels are high and releases the components when L-homocysteine is low and the demands for the amino acid are high (Lee et al., 2001). In addition there is evidence to suggest that non-specific alkaline phosphatases (ALPs) can also hydrolyse AMP to adenosine (Picher et al., 2003).

## ADENOSINE RECEPTORS

**FIGURE 2:**
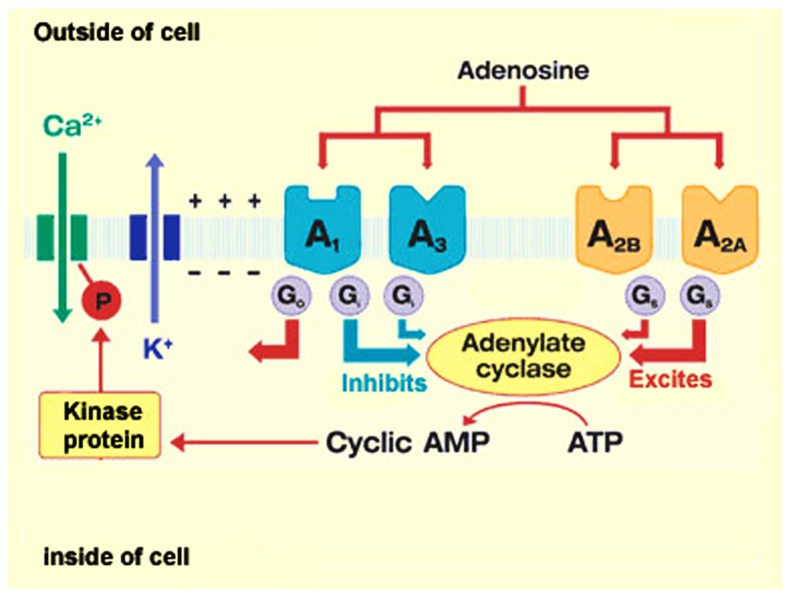
**Adenosine receptor signaling.** Adenosine mediates its action via four G-protein coupled receptors, A1, A2A, A2B, and A3 that are coupled primarily to the activation and inhibition of cAMP. There is also evidence to suggest that accumulated cAMP is linked to the modulation of ion-channel activity (reprinted from herbalzym.com).

Activation of ARs is dependent on the presence of extracellular adenosine which can bind to a family of four P1 or A receptors, termed A1, A2A, A2B, and A3. All four receptors are G-protein coupled, either Gs or Gi and signal primarily through the activation (A2A and A2B) or inhibition (A1 and A3) of cAMP (**Figure 2**). The four receptors have also been reported to activate phospholipase C and mitogen-activated protein kinase (Fredholm et al., 2001). There is strong homology between specific ARs in different species and all are asparagine-linked glycoproteins with seven transmembrane sequences. The A1 receptor comprises six exons (two of which are coding) and the other ARs have two coding exons (Olah and Stiles, 2000; Fredholm et al., 2001; Hasko et al., 2005). A1 and A2A receptors, in particular, can heterodimerize with D1 and D2 dopamine receptors (Kudlacek et al., 2003; Fuxe et al., 2007; Kim and Palmiter, 2008) and Group 1 and 2 metabotropic glutamate receptors (Di Lorio et al., 1996; Ferre et al., 1999) providing another level of functional control. There is also evidence to suggest that adenosine can interact or activate ion channels such as L- and N-type calcium channels (Mei et al., 1996), voltage-gated calcium channels (Chieng and Bekkers, 2001; McCool and Farroni, 2001) and K^+^ channels (Paisansathan et al., 2010). ARs are widely expressed, and have different affinities for adenosine – A1 and A2A receptors are to a certain extent high affinity receptors (*K*_m_< 30 nM) whereas the A3 and particularly the A2B receptor are low affinity receptors (*K*_m_ 1–20 μM). The A2B and A3 receptors are thus only likely to be activated under high metabolic and stressful cellular conditions (Spychala, 2000; Fredholm et al., 2001; Pedata et al., 2007).

## ADENOSINE METABOLISM AND BONE PHYSIOLOGY

It is plausible to suggest that enzymes involved in the ATP–adenosine metabolic pathway could influence bone physiology. A few years ago it was suggested that metformin, commonly used for the treatment of type II diabetes and known to act via activation of AMPK, may have an effect on bone (Kanazawa et al., 2008). In these studies the authors showed that metformin stimulated type 1 collagen and osteocalcin expression, ALP enzyme activity, and mineralization of MC3T3-E1 osteoblast cells. They also suggested that AMPK was the mediating molecule as the osteoblastogenic effects of metformin were reversed with specific AMPK inhibitors. Similar findings were found in studies using primary rat calvarial osteoblasts and the rat ROS 17/2.8 osteoblast cell line (Shah et al., 2010) and micro-computed tomography (μ-CT) studies showed that AMPα1^(^^-^^/^^-^^)^ knockout (KO) mice had smaller cortical and trabecular compartments than wild-type mice (Shah et al., 2010). In addition to its stimulatory effects on osteoblasts, AMPK also appears to mediate the inhibition of bone resorption by down-regulating the receptor activator of NF-κB ligand (RANKL) in osteoclasts (Lee et al., 2010; Jeyabalan et al., 2012). Animals with germline deletions of AMPKβ1 and 2 also had reduced trabecular bone density and mass compared with wild-type littermates, although there was no change in osteoblast or osteoclast cell numbers (Quinn et al., 2010). These results show that AMPK is important for maintaining homeostatic bone density but has no effect on osteoclast function or differentiation.

CD73, together with ADA are the primary enzymes involved in the generation and metabolism of adenosine. St. Hilaire et al. (2011) and Robson (2011) reported the existence of non-sense or missense mutations in the CD73 gene (resulting in a non-functional protein) in sibling members with lower extremity arterial calcification in three families. The authors hypothesize that reduced adenosine levels increases ALP activity and consequently increased degradation of pyrophosphate to inorganic phosphate which in turn affects arterial calcification. This would be of significance in the bone also as it is accepted that the bone mineralization process depends on the regulated balance of many factors including inducers such as inorganic phosphate and inhibitors such as inorganic pyrophosphate (Mackenzie et al., 2012). More recently, Takedachi et al. (2012) characterized the bone phenotype of cd73^-^^/^^-^ mice, and investigated the involvement of CD73 and AR signaling in osteoblast differentiation *in vitro*. Analysis by μ-CT showed that 13-week-old male cd73^-^^/^^-^ mice had osteopenia, and that this was due to reduced trabecular bone volume, decreased trabecular number and thickness, and increased trabecular separation when compared to wild-type controls. Other work measuring biochemical markers of bone formation showed that osteocalcin (bone formation marker) was decreased in cd73^-^^/^^-^ mice whereas bone resorption markers [tartrate-resistant acid phosphatase 5b (TRAP5b) and C-terminal telopeptide] were comparable to wild-type animals. Quantitative RT-PCR further substantiated the association of the osteoblast to the impaired bone phenotype seen in these animals. These studies suggest that the involvement of CD73 in bone homeostasis is directed to the osteoblasts. Other work included in the paper describes a series of *ex vivo* experiments using osteoblasts isolated from cd73^-^^/^^-^ and wild-type animals. The authors suggest that CD73 plays a role in osteoblast differentiation but not in the development of osteoblast progenitors. In addition, the authors describe experiments with MC3T3 (a mouse osteoblast cell line) cells and conclude that overall their work shows that CD73-generated adenosine positively regulates osteoblast differentiation via A2B AR signaling.

Skeletal abnormalities are also reported to be associated with disorders of the immune system such as ADA deficiency which may be related to increased osteoclast bone resorption through activation of cytokine molecules (Hong, 1989). Studies in ADA negative mice (ADA^(^^-^^/^^-^^)^) showed that they have reduced trabecular bone density and reduced RANKL but no change in osteoprotegerin (OPG; Sauer et al., 2009). The reduced bone turnover in ADA^(^^-^^/^^-^^)^ animals appears to be due to decreased osteoblastogenesis but little change in osteoclastogenesis. Interestingly, the plasma RANKL/OPG ratio was also significantly increased in ADA-deficient patients (Sauer et al., 2009). These data thus suggest that ADA is an important regulator of bone function by altering primarily osteoblastogenesis.

## ADENOSINE RECEPTORS AND BONE

The expression and function of ARs in bone cells were first demonstrated by Shimegi who showed that adenosine was mitogenic for the MC3T3-E1 osteoblast cell line (Shimegi, 1996, 1998). In addition methotrexate, a drug frequently used to treat rheumatoid arthritis and thought to mediate its actions via the release of adenosine (Chan and Cronstein, 2010), was shown to inhibit ALP activity in MC3T3-E1 and rat bone marrow stromal cells (Uehara et al., 2001). A seminal paper in 2006 showed that extracellular adenosine was produced by human osteoprogenitor and mesenchymal stem cells (MSCs; Evans et al., 2006). In addition, this study demonstrated the presence of the four AR subtypes (A1, A2A, A2B, A3) in these cell types and showed that adenosine and other AR agonists modulate the secretion of (i) the inflammatory cytokine IL-6 and (ii) OPG, a key modulator of osteoclastogenesis.

Since this finding, a flurry of key papers that support the hypothesis that ARs play a key role in bone homeostasis have been published. Thus we now have much more insight into the roles of these receptors in osteoblasts and osteoclasts as well as in their respective progenitor cells. To provide an overview of the literature in relation to current understanding, the next sections will discuss published work under separate headings for each of the AR subtypes. These sections will include work undertaken *in vitro* on cell lines and primary bone cells, as well as information gleaned from working with AR KO mice. Mouse models that harbor a deletion within each of the four ARs are now available for research purposes (Yaar et al., 2005; Yang et al., 2006), and these have led to significant conclusions on different AR function in many tissues. Significantly, in the last few years most of these have also been studied in relation to bone quality and density. Published studies on the role of the A3 AR in musculoskeletal tissues focus on arthritis so this will be discussed in Section “Adenosine and Adenosine Receptors in Arthritis.” We are not aware of any published studies that have explored bone density and quality in the A3 AR KO mouse.

### A1 ADENOSINE RECEPTOR

A1 ARs play important roles in promoting human monocyte fusion into giant cells *in vitro* (Merrill et al., 1997), and in inducing appropriate formation and function of osteoclasts (Kara et al., 2010a). Surprisingly, however, when similar work was undertaken using cells from A1 AR KO mice, although osteoclast formation was defective *in vitro*, these animals exhibited normal numbers of osteoclasts *in vivo* (Kara et al., 2010a,b). However, these osteoclasts *in vivo* did not seem to be actively resorbing bone. No morphological changes were observed in osteoblasts in these animals and bone-labeling studies did not reveal a change in bone-formation rates (Kara et al., 2010a). Consistent with these findings, there was a significant increase in bone density in the A1 AR KO mice, and administration of an A1 AR antagonist prevented ovariectomy-induced bone loss (Kara et al., 2010a). A recent paper by the same group (He and Cronstein, 2012) addressed the disparity between *in vitro* and *in vivo* osteoclast formation described above and postulated that key factors could be present *in vivo* but not *in vitro*. They thus further delineate the mechanisms by which the A1 AR modulates RANKL-induced signaling in osteoclastogenesis and conclude that the receptor regulates osteoclast formation by altering tumor necrosis factor-receptor-associated factor/transformation growth factor-β activated kinase-1 (TRAF/TAK1) signaling (He and Cronstein, 2012). Pellegatti et al. (2011), however, show very little expression of the A1 AR in quiescent or macrophage-colony stimulating factor (M-CSF)/RANKL-stimulated human monocytes and state that the A1 AR does not play a role in osteoclastogenesis. They hypothesize that the differences between their observations and those of others (Merrill et al., 1997; Kara et al., 2010a,b) are due at least partially to species difference.

Although it is known that A1 ARs are expressed in osteoblasts, their roles are largely unknown. We (Gharibi et al., 2011a,b) have recently shown that A1 AR expression and function in MSCs and mouse 7F2 osteoblasts seemed to be more relevant to adipocyte differentiation rather than osteoblast differentiation. When MSCs and 7F2 cells were induced to differentiate to adipocytes, the increased expression of adipocyte marker genes such as CCAAT enhancer binding proteins β (C/EBPβ) and lipoprotein lipase (LPL) was accompanied by significant increases in A1 AR expression. In support of these findings stable transfection of the human A1 AR gene into 7F2 osteoblasts increased the expression of LPL but decreased expression of ALP (Gharibi et al., 2011b). Over expression of the A1 AR also enhanced lipid accumulation as determined by oil red O and nile red labeling (Gharibi et al., 2011b).

### A2A ADENOSINE RECEPTOR

It has been reported that A2A ARs play an important role in promoting human monocyte fusion into giant cells *in vitro* (Merrill et al., 1997). The same group has very recently extended their studies and shown that A2A AR ligation inhibits osteoclasts formation (Mediero et al., 2012a). This work demonstrated that the A2A AR agonist, CGS 21680, inhibited osteoclast differentiation and function, increased the percentage of immature osteoclast precursors, and decreased IL-1β and tumor necrosis factor-α (TNF-α) secretion. Cathepsin K and osteopontin mRNA expression increased in control and ZM241385 (A2A AR antagonist) pretreated osteoclasts, and this was blocked by CGS 21680. Furthermore, μ-CT analysis of A2A AR KO mouse femurs showed a significantly decreased bone volume/trabecular bone volume ratio, decreased trabecular number, and increased trabecular space. These A2A AR KO mouse femurs also showed an increased number of TRAP-positive osteoclasts, with marked osteoclast membrane folding and increased bone resorption.

It is well accepted that P2 purinergic receptors (especially the P2X7 receptor) are involved in osteoclast formation and function. It has recently been shown (Pellegatti et al., 2011) that the P2X7 receptor, ATP, adenosine, and ARs act in a concerted fashion to promote fusion of M-CSF/RANKL-stimulated monocytes into multinucleated osteoclasts. This work also showed that A2A AR antagonism potently inhibited fusion, whilst CGS 21680, an A2A agonist, potentiated fusion but was able to overcome P2X7 receptor blockade. Some of these observations are opposite to those described by others (Mediero et al., 2012a) and a very recent manuscript from the Cronstein group further disagrees with these observations as it demonstrates a direct role of A2A ARs in human osteoclasts formation (Mediero et al., 2012b), i.e., results which tie in with what they have previously shown in the mouse (Mediero et al., 2012a).

A role for the A2A AR in bone homeostasis is further supported by observations indicating that in a collagen-induced mouse model of arthritis significantly less bone resorption was observed in the CGS 21680-treated mice when compared to sham-control and sham-CGS 21680 mice (Mazzon et al., 2011). These observations are in agreement with other studies described above using *in vitro* cell systems or A2A KO mice.

In relation to the osteoblast lineage, it has been shown that the A2A AR plays a role in mouse bone marrow-derived MSC development (Katebi et al., 2009). The authors conclude that adenosine and the A2A AR play a critical role in promoting the proliferation and differentiation of mouse bone marrow-derived MSCs. It has also been shown using rat-derived MSCs, that the expression of A2A AR was upregulated during later stages of osteoblastic differentiation, when its activation stimulated ALP activity (Gharibi et al., 2011a).

### A2B ADENOSINE RECEPTOR

The A2B AR is expressed in human osteoclast precursors (quiescent monocytes) and in those activated with M-CSF/RANKL (Pellegatti et al., 2011). Furthermore the same study did not demonstrate an effect when an A2B AR antagonist was added to an osteoclast formation assay. We are not aware of any further information currently available relating to A2B AR expression and function in osteoclast differentiation and activity.

There is, however, strong evidence for the involvement of the A2B AR in osteoblast differentiation and function. It has been shown that this receptor is functionally dominant in human osteoprogenitor cells (Evans et al., 2006). It has also been shown that the A2B AR was dominant in rat bone marrow-derived MSCs, and its expression and activity were transiently upregulated at early stages of osteoblastic differentiation. Both activation and overexpression of A2B AR induced the expression of osteoblast-related genes (runx2, ALP), as well as ALP activity, and stimulation increased osteoblast mineralization (Gharibi et al., 2011a). Others (Takedachi et al., 2012) have also concluded that the A2B AR is important in osteoblast differentiation. Blockade of the A2B AR with PSB603 prevented osteogenic differentiation when human primary MSCs were stimulated to differentiate to osteoblasts in the presence of the universal AR agonist (NECA; Costa et al., 2011). This work also shows that A1, A2A, A2B, and A3 AR agonists increased human primary osteoblasts proliferation, and that this agonist-induced proliferation was prevented by a panel of receptor antagonists specific for each of the four receptor subtypes (Costa et al., 2011). This is in contrast to the work of others (Evans et al., 2006) who report that NECA (AR universal agonist) did not change cell numbers when using a human osteoprogenitor cell line, even though the concentrations used were similar in both studies.

Other work published this year (Carroll et al., 2012) focuses on the differentiation of mouse MSCs to osteoblasts. The authors build on what others have shown using *in vitro* pharmacological approaches by investigating the role of the A2B AR in osteoblast differentiation and function both *ex vivo* and *in vivo* using A2B AR KO mice. *Ex vivo* studies showed that at 9 and 12 days after osteo-induction, bone marrow from these KO mice showed fewer mineralized nodules, demonstrating a reduction in osteoblast differentiation. They also examined the expression of transcription factors central to osteoblast differentiation, runx2 and osterix, and found that with osteo-induction, the increase in the expression of both runx2 and osterix was significantly attenuated in the A2B AR KO samples. Other studies involving activation of the A2B AR or addition of a cAMP analog during differentiation led them to conclude that the mechanism of effect involves, at least partially, cAMP.

Carroll et al. (2012) also addresses the role of the A2B AR in callus formation after fracture. μ-CT revealed that fracture calluses of A2B AR KO mice showed a smaller overall total volume, as well as a decrease in the ratio of bone volume to total callus volume at 14 and 21 days post-fracture. Analysis of tissue sections supported these findings. In addition, tissue from the fracture callus in A2B AR KO mice tend to show decreased expression of runx2 and osterix at 3 days post-fracture and showed significantly decreased expression at 7 days post-fracture, as compared to wild-type animals. The authors conclude that such findings suggest that the A2B AR is involved in aspects of the skeletal repair process and its absence leads to delayed progression of bone development during fracture repair in the A2B AR KO mice. Furthermore, and unexpectedly, analysis of adult femurs showed lower bone density in A2B AR KO mice as compared to wild-type animals, and the A2B AR KO animals have significantly shorter femurs.

## ADENOSINE AND ADENOSINE RECEPTORS IN ARTHRITIS

Adenosine is known to be released under tissue injury or cellular stress and high concentrations (18–50 μM) have been found in the synovial fluid of rheumatoid arthritis patients (Sottofattori et al., 2001; Huang et al., 2004). The accumulation of adenosine in the synovial fluid could be due to inhibition of the AK enzyme; the effects of such an inhibitor, ABT-702 (a selective non-nucleoside AK inhibitor) have been investigated in the rat adjuvant arthritis model (Boyle et al., 2001). Animals treated with ABT-702 showed reduced cartilage and bone destruction, and an overall inhibition of paw volume and hence arthritis. These data would thus suggest that adenosine plays a beneficial role in bone formation. One of the earliest reports that suggested a possible involvement of adenosine in bone pathology in arthritis used the collagen-induced arthritis model in mice, where administration of Z-5′-fluoro-4′,5′-didehydro-5′-deoxyadenosine (MDL 28,842 an irreversible inhibitor of SAH hydrolase) had fewer bone lesions than control animals (Wolos et al., 1993). Inhibitor treated animals also showed a decrease in the incidence of arthritis as well as a delay in disease development.

Further studies in murine MC3T3-E1 osteoblast cells showed that adenosine can protect against cell death induced by hydrogen peroxide and these protective effects could be mediated via A1 and A2A ARs (Fatokun et al., 2006). The actions of adenosine on bone in the setting of rheumatoid arthritis could be due to its anti-inflammatory properties; the A2A AR agonist, CGS 21680, ameliorated arthritis in the collagen-induced mouse by reducing bone resorption and inhibiting plasma concentrations of IL-6, soluble IL-1 and TNF-α as well as nitric oxide synthase and cyclooxygenase-2 (Mazzon et al., 2011). In recent years there has been a focus on the possible use of A3 AR agonists as therapeutic compounds in rheumatoid arthritis; IB-MECA (A3 AR agonist) reduced pannus and fibrosis formation, cartilage and bone destruction, and the latter via a decrease in the number of osteoclasts in the adjuvant mouse model (Rath-Wolfson et al., 2006). In these experiments, IB-MECA also appeared to down-regulate A3 AR expression and several inflammatory related molecules (e.g., AKT, NFκB, TNF-α, and RANKL).

Similar findings were found when the highly selective A3 AR agonist IB-MECA (CF101) was given orally to rats that had been induced to develop osteoarthritis with monosodium iodoacetate (Bar-Yehuda et al., 2009). The A3 receptor selective antagonist MRS1220 reduced the beneficial effects (inhibition of bone destruction and down-regulation of NFκB and TNF-α) of CF101. There is also evidence to show that the actions of methotrexate, the mainstay drug for rheumatoid arthritis, are mediated through activation of ARs. For example, in the case of methotrexate-induced expression of the A3 receptor, addition of CF101 with methotrexate enhances the effect of the AR antagonist (Ochaion et al., 2006). The non-specific AR antagonists, theophylline and caffeine reversed the effect of methotrexate on hindpaw swelling and ankylosis in experimental adjuvant arthritis (Montesinos et al., 2000). Methotrexate also mediates its actions via the transcription factor NURR1; such actions could also be mimicked by adenosine and its stable analog, NECA, suggesting that methotrexate may stimulate adenosine release (Ralph et al., 2005). *In vitro* studies have also suggested that methotrexate, in the context of rheumatoid arthritis, has an inhibitory action on osteoclastogenesis which is abolished by adenosine acting through the A2B AR. Furthermore adenosine injected into the ankle joints of methotrexate-treated adjuvant arthritic animals suppressed the effect of methotrexate on bone destruction (Teramachi et al., 2011). These data may suggest that adenosine has a stimulatory effect on osteoclastogenesis.

## ARs AS THERAPEUTIC TARGETS

Adenosine receptors have long been considered to be possible therapeutic targets in a wide range of conditions including cardiac, pulmonary, immunological, and inflammatory disorders (Fredholm, 2003, 2010; Jacobson and Gao, 2006). Medicinal chemistry approaches have led to the development of selective and potent agonists and antagonists for each of the four AR subtypes (Muller and Jacobson, 2011), and many such compounds have been, or are currently, in clinical trials. For example, IB-MECA (CF101, developed by Can-Fite BioPharma), an A3 AR agonist, was recently demonstrated to be efficacious in clinical trials of rheumatoid arthritis, psoriasis and dry eye disease (http://www.canfite.com) and further clinical trials are planned for osteoarthritis and glaucoma (Muller and Jacobson, 2011).

Inhibitors of nucleoside metabolism also represent an alternative therapeutic strategy that has gained increasing attention in recent years (Boison, 2011). The augmentation of nucleoside function by inhibiting their metabolism has several advantages including the potential of affecting several signal transduction pathways simultaneously and may thus be suitable for fine-tuning, restoring, or amplifying the physiological functions of adenosine. Of special interest also is the development of radioligands for *in vivo* imaging of ARs for diagnostic use in the CNS and in the periphery. Ligands for *in vivo* positron emission tomographic (PET) imaging of A1, A2A, and A3 ARs have been developed to date and it will be interesting to see whether they will play a role in furthering our knowledge of ARs in musculoskeletal tissues. Generally speaking, however, we need to accumulate more data of the mode of action of ARs in human bone before any of the AR agonists and antagonists currently available are used in bone disease related clinical trials.

## CONCLUSION

It is clear from the work reported here that there is currently strong evidence to support a role for adenosine and its receptors in bone homeostasis, and these signaling pathways could have significant relevance in bone diseases such as osteoporosis and arthritis. There have been major advances in the field in recent years, but there is much more to learn. Most of the studies to date utilize cells (cell lines and primary) *in vitro*, or animal models *ex vivo* and *in vivo*. Studies using human material are thus required urgently to complement what is already known. As our knowledge of adenosine metabolism and signaling pathways accumulates, we will also hopefully move toward developing new therapeutic strategies for bone disease.

## Conflict of Interest Statement

The authors declare that the research was conducted in the absence of any commercial or financial relationships that could be construed as a potential conflict of interest.
